# Building better microbial infection models: a call to do the “field” experiments

**DOI:** 10.1128/mbio.01768-26

**Published:** 2026-08-24

**Authors:** Marvin Whiteley

**Affiliations:** 1 School of Biological Sciences, Georgia Institute of Technology 123387, Atlanta, Georgia, USA; 2 Emory-Children’s Cystic Fibrosis Center, Atlanta, Georgia, USA; NYU Langone Health, New York, New York, USA

**Keywords:** model systems, biofilm models, host-microbe interactions, polymicrobial communities, transcriptomics

## Abstract

Experimental model systems are essential in microbiology. However, models are often described as “biologically relevant” without a clear explanation of what that means or how relevance was established. Here, I argue that the missing piece in model development is benchmarking. New technologies and increasingly elaborate model systems can be powerful, but they do not guarantee that a model better represents the environment it is meant to reproduce. The guarantee that is implied cannot be made explicit until these models are evaluated against measurements of microbial behavior and function made directly in those environments. These “field” experiments are often messy, expensive, low throughput, and technically challenging, but provide the benchmarks needed to determine what a model captures, what it misses, and which questions it can tackle. While chemical measurements and quantification of physical features can guide model construction, the most important readout is the behavior of the microbes in the natural environment.

## PERSPECTIVE

Microbiologists rely on experimental models. This is true whether we study microbes in the soil or the ocean, biofilms on medical devices, or microbial communities in and on the human body. Since natural environments are often complex, variable, and difficult to manipulate, model systems allow us to simplify this complexity, control variables, perform perturbations, and test mechanisms. Without models, much of microbiology would remain descriptive. But experimental models also create a problem. A model is useful only to the extent that it captures the biology relevant to the question being asked. A model system can be reproducible and experimentally powerful without accurately reproducing the microbial behaviors that occur in the natural environment. Yet models are often described as “biologically relevant” without a clear explanation of what that means or how relevance was established. In the past, I have made this claim myself. The central challenge is not building model systems. It is model accountability: stating what a model is supposed to test and showing that it does.

This perspective focuses on experimental models used to study host-associated microbes, especially those relevant to human infection. These systems provide clear examples of the challenges involved in model development because the native environments are often difficult to access, chemically and spatially complex, and often polymicrobial. However, the argument I am making extends beyond infection biology. The same challenge applies to models of soil, plants, marine, wastewater, and other microbial communities.

Microbiology has made major progress in developing model systems that better approximate natural environments. Artificial media, microcosms, biofilm systems, tissue models, animal models, organoids, microfluidic devices, and synthetic communities have all been used to study microbes under presumably more realistic conditions (1–5). This progress should not be diminished. In cystic fibrosis (CF) infection research, for example, many groups have developed model systems to study chronic airway infections (3, 6–11). These models have been essential for understanding how pathogens behave in the CF lung. Similar efforts have shaped studies of wounds, oral biofilms, the gut microbiome, plant-associated communities, and environmental microbial communities. The question is not whether these models are valuable. They clearly are. The question is, how do we determine which aspects of the natural environment they reproduce?

This question is often harder to answer than it seems. Models are commonly judged by practical or visible features, including, among other things, whether they are reproducible, support growth, include particular environmental components or microbial species, or use technically impressive platforms. These can of course be useful features, but they do not prove that the model reproduces the biology of the natural environment. This distinction matters. For example, a model can be technically impressive or include many species found in an environment and still fail to capture the physiology of the microbes in the environment it is meant to represent. Complexity is not the same as accuracy.

**Fig 1:**
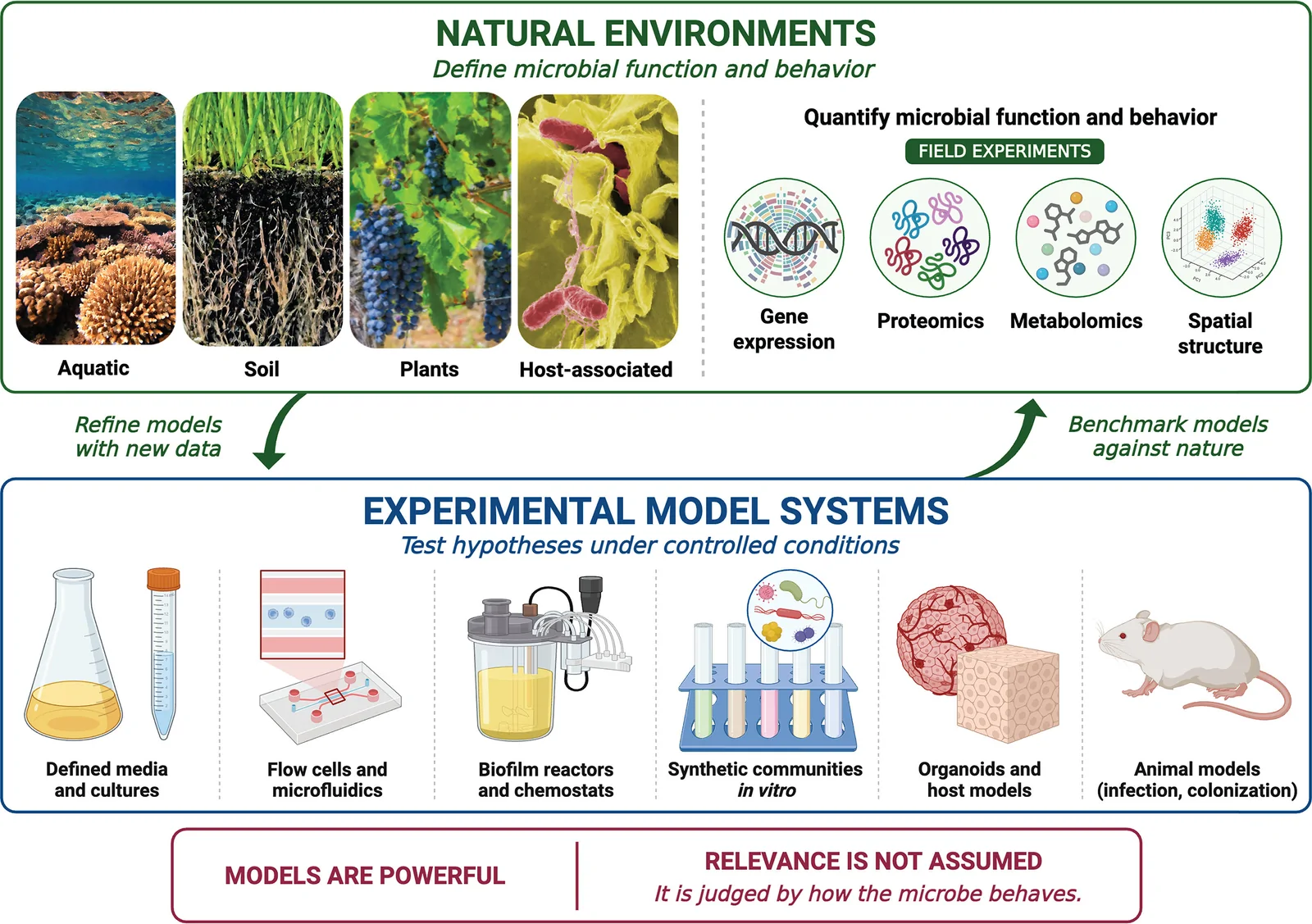
Benchmarking model systems. Field experiments using samples from natural environments define the microbial functions and behaviors that experimental models should reproduce and provide quantitative benchmarks for evaluating and refining those models.

I propose that the missing piece is benchmarking (Fig. 1). By benchmarking, I mean comparing microbial behavior in a model to microbial behavior in the natural environment the model is meant to represent (8, 12, 13). The goal is often not to reproduce every feature of that environment. That is generally not possible, and it is not necessary for tackling many questions. Instead, it is important to define what biology the model captures, what it misses, and which questions it can reasonably answer. This requires doing the “field” experiments, which directly assess microbial function and behavior in the natural environments we are trying to model. These experiments are often messy, low throughput, expensive, and technically challenging to analyze. They are affected by sample-to-sample variation, contamination, complex microbial communities, and limited material. But they are essential because they provide the data needed to judge how closely a model reproduces that environment. Importantly, the appropriate benchmark depends on the biology being modeled. Population-level benchmarking is useful because it identifies conserved microbial behaviors, but it may average out transient or subpopulation-specific phenotypes present in dynamic ecosystems. If the goal is to model a particular ecological state or minor subpopulation, then the benchmark should be designed at the single-cell or subpopulation level rather than the population average. Benchmarking should also be a community effort: public transcriptomic, proteomic, metabolomic, imaging, and spatial data sets can serve as shared references for groups that cannot generate field data themselves.

There are many ways to design a model. One approach is to measure the chemistry of the natural environment (10, 14–16). What nutrients are present? Which electron acceptors are available? What is the pH? How much oxygen is present? These measurements are valuable because they help guide the chemical composition of an experimental model. However, in my opinion, they are not enough, as they do not tell us whether the model has recreated conditions that induce natural behaviors in the microbe. To answer that question, we have to ask the microbe.

Microbes are highly sensitive to the composition of their environment, and their responses to those conditions shape their behavior. These responses often integrate many features of the environment at once and are often not predictable. For this reason, I propose that microbial behavior is often the best readout of whether a model captures the conditions that matter. Our intuition and knowledge about a natural environment can guide model development, but it should not be the benchmark. The benchmark should be a quantitative measure of how the microbe behaves in its natural environment, and the model is only realistic when it recapitulates these behaviors.

In our own work, we have often used bacterial RNA sequencing to query microbial behavior in their natural environments for benchmarking (6, 8, 12, 13, 17, 18). By measuring gene expression directly in these settings, we can infer what functions are present and then ask whether those same functions are reproduced in laboratory models. When we started doing these experiments, they were sometimes dismissed as fishing expeditions. In some sense, that criticism was correct. We were fishing, but we were fishing where the bacteria live. The objective was not simply to describe microbial gene expression during human infection but to use these data to infer the functions and behaviors important to reproduce in a model.

As one example, we compared *Pseudomonas aeruginosa* gene expression in human infections to that in laboratory conditions (12, 13, 17), then asked which models promoted *P. aeruginosa* gene expression patterns most similar to those observed during human infection. These studies revealed that no model was perfect, but that different models captured different aspects of human infection physiology and therefore had different uses. They also showed that mismatches in gene expression between models and human infections can be informative. For example, when *P. aeruginosa* gene expression in a model does not match gene expression in the human infection, the mismatch can point to specific ways the model can be changed (Fig. 1). We have used these mismatches in gene expression to identify missing features in a model, such as a need to restrict zinc levels in *P. aeruginosa* infection models (8). In this way, these field experiments using human infection material provided direction. They tell us what the model needs to include to induce the bacterial behaviors and functions found in the natural environment.

RNA sequencing is only one way to query bacterial function and behavior in natural environments. Proteomics, metabolomics, microscopy, spatial transcriptomics, isotope labeling, and genomics can potentially serve as benchmarks. The right benchmark depends on the question being asked. If different benchmarks disagree, the most important readout should be the one most directly tied to the biological question the model is being used to address. Models of polymicrobial infection provide a key example. Many natural environments contain multiple microbial species, and there is strong interest in building models that include this complexity (3). However, adding species together in the lab does not automatically make a model more relevant or valuable. A community may contain the right organisms but still have the wrong physiology, spatial organization, or interactions. Therefore, polymicrobial models need to be benchmarked at the level of behavior, not simply composition and co-existence. Are the same pathways active? Are the organisms using similar nutrients? Are they experiencing similar stresses? Are they spatially organized in similar ways? These are the types of questions that determine whether a polymicrobial model is relevant to the natural environment.

This does not mean that every model must be highly complex. Benchmarking may show that a simple model is sufficient for a specific question. A single bacterium growing in a simple *in vitro* medium may reproduce the relevant physiological state better than a complex lab-on-a-chip or organoid model. Simply using a non-laboratory strain in a model may significantly increase relevance of the model. In other cases, more complex systems may be required. The point is that model complexity should be justified by the biology being modeled, not by the assumption that more complexity is better. This also means that it is unlikely that a single model will be best for everything. One model may be good for studying nutrient limitation. Another may be better for antibiotic tolerance. This is not a problem. It is how models should work.

It is also important to point out that some models are not intended to reproduce the natural environment at all. They are built to define principles. Years ago, my laboratory collaborated to develop microfabricated devices that we called bacterial lobster traps (19, 20). These devices confined *P. aeruginosa* and other bacteria in picoliter-scale “houses” at defined distances from one another, allowing us to determine the spatial scale over which cells can detect neighboring cells. The goal was not to recreate the CF lung, a chronic wound, or any other *P. aeruginosa* native environment but to identify the spatial scale over which bacterial interactions could occur. In that case, the value of the model was not that it mimicked infection. Its value was that it provided guideposts for thinking about where and how to look for interactions in more complex infection environments. Thus, some models provide guideposts for testing fundamental principles, while others are intended to reproduce specific features of natural environments. Problems arise when we confuse these objectives. A reductionist model can be extremely powerful, but it should not be assumed to represent a native environment unless that connection has been tested.

We should be explicit about the intent of our models. When we develop or use a model, we should state what the model is intended to do. Is it designed to define a principle, isolate a variable, identify a scale, or reproduce a feature of a natural environment? If it is meant to represent a natural environment, then we should state what environment it represents, which feature of that environment it is designed to capture, what native measurements support the model, and what important features are missing. This would not impose a rigid standard for all studies. A simple model accountability checklist would ask, (i) what is the model intended to do, (ii) what benchmark supports it, (iii) what does it capture and what does it miss, and (iv) which conclusions require caution?

In summary, I propose that we need better-defined and better-benchmarked models as much as we need new ones. Clean laboratory systems and dirty field experiments should not be viewed as competing approaches. They are both necessary. Reductionist models can define principles and mechanisms. Field experiments are valuable fishing expeditions as long as they tell us what microbes are doing in the real world. Finally, when we are tempted to describe a model as biologically relevant, we should first ask what we mean by that statement. Relevant for which organism, which environment, which behavior, and which question? To answer these questions, we need to do the field experiments.
